# A step in the right direction: an open-design pedometer algorithm for dogs

**DOI:** 10.1186/s12917-018-1422-3

**Published:** 2018-03-20

**Authors:** C. Ladha, Z. Belshaw, J. O’Sullivan, L. Asher

**Affiliations:** 1 Centre for Behaviour and Evolution, Henry Wellcome Building, Newcastle University, Newcastle, NE2 4HH UK; 2 VetSens. 53 Wellburn Park, Jesmond, Newcastle, NE2 2JY UK; 3 School of Veterinary Medicine and Science, University of Nottingham, Sutton, Bonington Campus, Leicestershire, LE12 5RD UK

**Keywords:** Step counting, Accelerometer, Dog, Motion analysis, Activity level, GPS

## Abstract

**Background:**

Accelerometer-based technologies could be useful in providing objective measures of canine ambulation, but most are either not tailored to the idiosyncrasies of canine gait, or, use un-validated or closed source approaches. The aim of this paper was to validate algorithms which could be applied to accelerometer data for i) counting the number of steps and ii) distance travelled by a dog.

To count steps, an approach based on partitioning acceleration was used. This was applied to accelerometer data from 13 dogs which were walked a set distance and filmed. Each footfall captured on video was annotated. In a second experiment, an approach based on signal features was used to estimate distance travelled. This was applied to accelerometer data from 10 dogs with osteoarthritis during normal walks with their owners where GPS (Global Positioning System) was also captured. Pearson’s correlations and Bland Altman statistics were used to compare i) the number of steps measured on video footage and predicted by the algorithm and ii) the distance travelled estimated by GPS and predicted by the algorithm.

**Results:**

Both step count and distance travelled could be estimated accurately by the algorithms presented in this paper: 4695 steps were annotated from the video and the pedometer was able to detect 91%. GPS logged a total of 20,184 m meters across all dogs; the mean difference between the predicted and GPS estimated walk length was 211 m and the mean similarity was 79%.

**Conclusions:**

The algorithms described show promise in detecting number of steps and distance travelled from an accelerometer. The approach for detecting steps might be advantageous to methods which estimate gross activity because these include energy output from stationary activities. The approach for estimating distance might be suited to replacing GPS in indoor environments or others with limited satellite signal. The algorithms also allow for temporal and spatial components of ambulation to be calculated. Temporal and spatial aspects of dog ambulation are clinical indicators which could be used for diagnosis or monitoring of certain diseases, or used to provide information in support of canine weight-loss programmes.

## Background

The ability to make objective measurements of physical activity is of clinical importance for many movement related disorders and to understand lifestyle risk factors for disease. As an example, osteoarthritis is estimated to affect 20% of adult dogs [1]. Affected dogs have changes in mobility, including reduced willingness to exercise, and a tendency to stop on walks [2]. Assessing changes in mobility forms a core part of monitoring treatment response in this disease. However, current outcome measures for this purpose are frequently un-validated and at risk of bias [3]. The few widely available outcome measures that are validated to assess canine mobility are reliant on owner observations that may be subjective or inaccurate [3, 4]. To solve this issue, researchers are turning to accelerometer based technologies that have the potential to objectively measure such movement based parameters. In addition to monitoring movement related disorders, movement based parameters could also monitor general levels of physical activity or energy exertion. Lack of physical activity is an important lifestyle risk factor, both directly, and indirectly through contributing to obesity, for a wide variety of diseases and behaviour problems [5]. In order to facilitate monitoring of physical movement parameters, here we present an open-design algorithm for monitoring canine steps (number and distance).

Accelerometer based activity monitors have been steadily gaining popularity and are generally available as either research-grade or consumer-based devices. Over the past decade, researchers have started to experiment with their suitability for animal populations, including domestic dogs (*Canis familiaris*). Particular focus has been given to measuring Physical Activity as a proxy for dysfunction or disease [6–10]. Research grade devices such as such as the Actical (Philips Respironics, Netherlands) and GT3X (Actigraph Inc., USA) (used in [11, 12] respectively) are attractive as they have software that facilitates data export in formats suitable for post-processing. Unfortunately, the parameters calculated are optimised for human measurements and parameters such as rest, calories and steps are invalid for canine subjects. This leaves the researcher to either make coarse mappings (as in [12]), or export raw data and develop their own species-specific algorithms. More recently, consumer based devices tailored for canine Physical Activity have emerged [13–19]. However, studies such as [20] which examine robustness, reliability and validity are limited. As the devices are tailored for consumers, management and extraction of bulk data from such devices (which is necessary for research studies) is often manual and cumbersome (e.g. using provided Apps). Most of the consumer devices are collar based and despite recognition of the influences of fitting and placement [21–23], these aren’t standardised between devices. Currently neither research or consumer based products targeted at dogs offer clear, characterized and detailed explanations of underlying algorithms to calculate parameters such as activity, sleep, step-counts or behaviour. This means before use in research, validation processes must be carried out. Furthermore, there is the possibility of further re-validation requirements following changes to algorithms or calculation approaches between firmware updates or across device versions or models. To mitigate these issues, some animal researchers have made attempts to fill gaps between dog-specific consumer devices and research-grade human devices by making their own sensors [10, 24]. This step is challenging and is a barrier to most.

Akin to humans, some of the most common diseases and welfare concerns have exercise-related outcome measures. Walking forms an important part of exercise for dogs [5] and thus a step counting measure has promise to be a suitable replacement for otherwise complex actigraphy signal, which measures gross motor activity. Step counts may be a more precise alternative to gross motor activity because they don’t include energy resulting from stationary behaviours like scratching or shaking. Furthermore, gross activity and step counts could be used synergistically to partition energy expended through walking separate to activities such as playing or tail chasing. Some dogs might engage in more play at home and expend more energy this way than out on walks which would not be apparent from the gross activity measures alone, but could be if combined with step count data. In humans, providing tools to monitor both cumulative [25] and temporal distribution of step counts [26], as well as the variability of step frequency and length of any one bout of walking, have proved useful in improving health outcomes. We hypothesise similar benefits could be realised in the domestic dog population, and thus an accurate tool for detecting steps and measuring their distance is important.

In this paper, a pedometer algorithm is described that is adapted specifically for the quadruped gait cycle and is optimised for canine gait. Measuring canine gait has different requirements to measuring human gait, both due to positioning of device and differences between the way human and dog gait is modelled. Our twofold aim was to develop a method for: (i) Counting steps; and (ii) Estimating distance travelled. The methods were designed to be suitable for quadrupeds, and particularly the domestic dog. The intention behind developing these methods was that they would replace existing methods that are: closed source [13, 14, 16, 19]; offer uncharacterised performance [27]; or are optimised for capturing human gait [28].

To count steps, an approach based on partitioning acceleration was used. We used data from an experimental cohort of healthy dogs (described further in [29]). Particular consideration was given to algorithms to only detect continuous forward locomotion; it rejects stumble or shuffles. The performance of the proposed algorithm was characterised by comparing step estimates from algorithms to those made through manual annotations of video footage.

To estimating step length, an approach based on signal features was used. This method was evaluated in a second experiment where walks were measured using a GPS system in a free-living setting (on- and off- lead walks without intervention) in a clinically relevant population (dogs with osteoarthritis). This sample of dogs was chosen for convenience and were recruited as part of a larger study and is described in more detail in [2]. The performance of the proposed algorithm was characterised by comparing estimates of walk length from algorithms to those made using GPS data.

## Methods

### Subjects - step counting

For the step count experiment a convenience sample of 13 healthy adult dogs were recruited from a local demographic using posters and local advertising. The dogs represent a range of breeds, sizes and ages of dogs, as well as being roughly sex balanced (see Table 1). Ethical approval was obtained from the animal welfare and ethical review body at Newcastle University for recruitment, exclusion and data collection processes. The sample contained breeds and crossbreeds of: Cocker Spaniel, Welsh Corgi, Doberman, Irish Wolfhound, Jack Russel Terrier, Labrador (*n* = 2), Lurcher (*n* = 3), Poodle, Rhodesdian Ridgeback and Springer Spaniel. All sections of this report adhere to the ARRIVE Guidelines for reporting animal research [30]. A completed ARRIVE guidelines checklist is included is Checklist S1.

**Table 1:** Details of subjects, steps detected using algorithm applied to accelerometer signal, and counts detected from video footage

ID	Height (cm)	Weight (kg)	Breed	Age	Sex^a^	Steps Counted Video	Steps Detected Algorithm
D.1	47.4	17.5	Springer Spaniel	9	FN	455	398
D.2	51.5	27	Mixed	8	FN	351	309
D.3	54	28.1	Labrador	5	FN	348	307
D.4	50.5	28.2	Mixed	10	FN	313	274
D.5	53.4	18.1	Lurcher	6	MN	311	269
D.6	60.6	39	Labrador	2	MI	275	242
D.7	45.8	11.3	Lurcher	1.9	F	323	293
D.8	64.4	25	Lurcher	9	MN	331	305
D.9	46.8	12	Springer Spaniel/Collie	5	FN	414	370
D.10	84.9	64.0	Irish Wolf hound	5.5	FN	274	274
D.11	38	11.8	Corgi	3.5	FN	491	455
D.12	44.4	15	Cocker Spaniel/Poodle	2	MN	489	374
D.13	61.5	38	Rhodesian Ridgeback	7	FN	320	281

^**a**^For sex *F* Female, *M* Male, *N* neutered

### Experiment- step counting

**Fig. 1:**
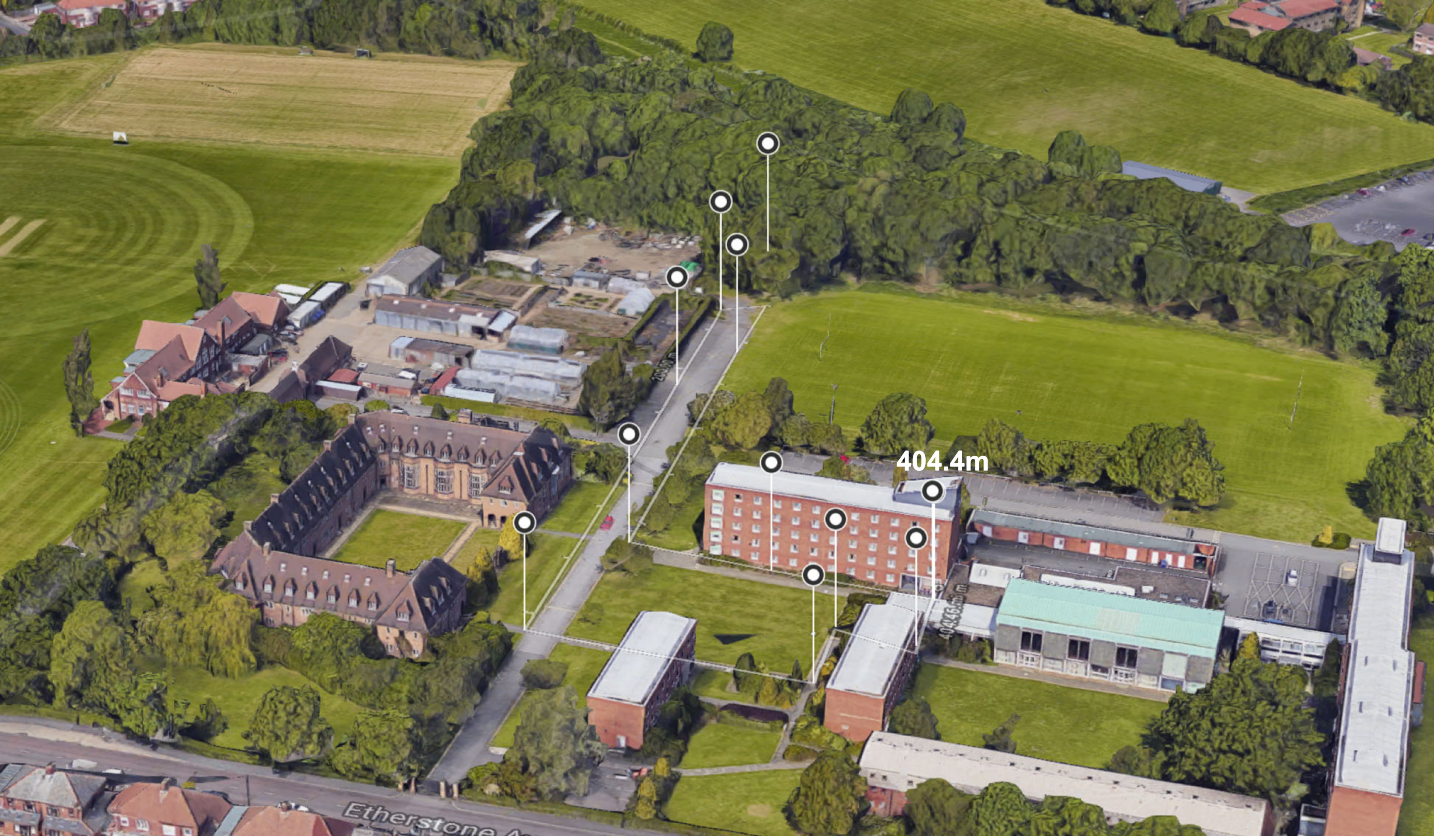
Image of the course used for walking the dogs during step counting experiment. (Image reproduced with permission Google ©2017)

**Fig. 2:**
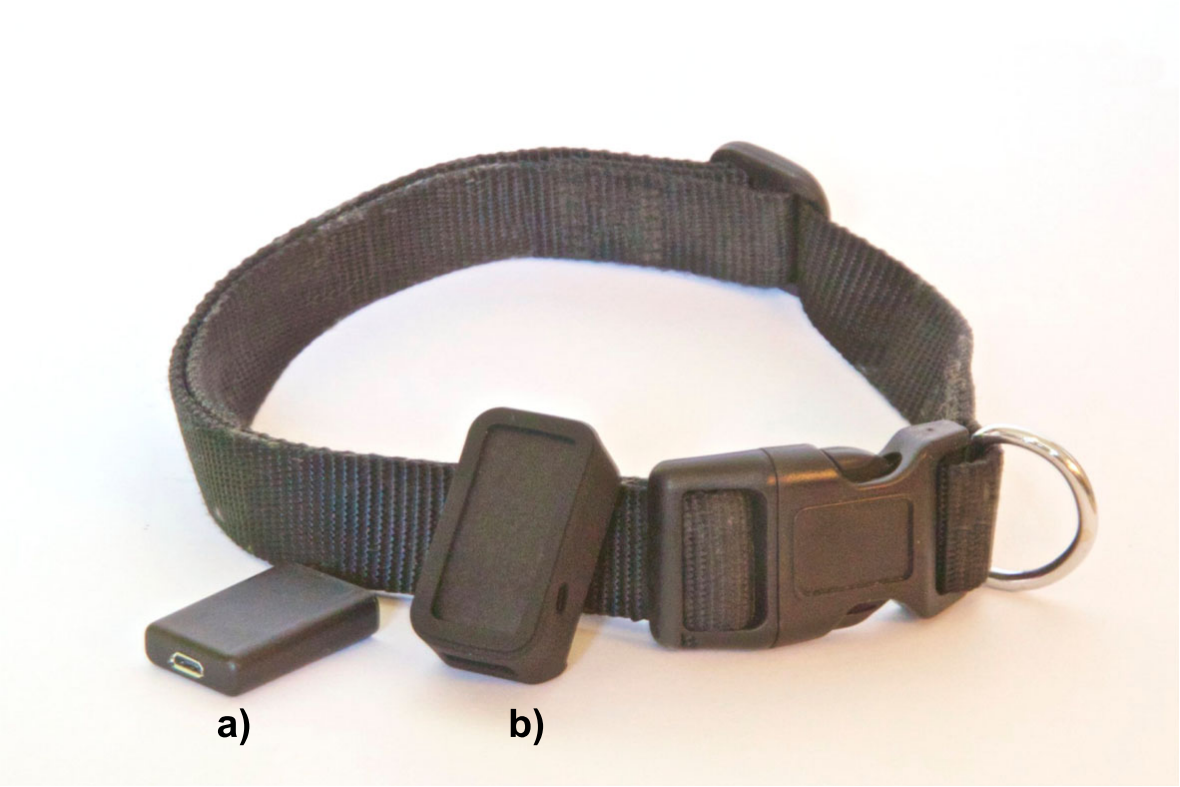
Photo of the accelerometer (**a**) and GPS sensor (**b**) alongside the collar used for collecting data

Each dog was walked around a set route of approximately 400 m (measured on Google Earth software v7.1) along a flat mix of tarmac and grass (see Fig. 1). Walks were videoed using a smart phone at 60 frames-per-second (iPhone 7, Apple Inc, USA) to enable subsequent manual counting of steps. At the start of each walk the dog was fitted with and habituated to a soft-weave, nylon, flat collar fitted with an accelerometer (VetSens, UK). The accelerometer was attached (as in Fig. 2) to the outside of the collar using a single layer of Gorilla Tape (Gorilla Glue Company, USA). The accelerometer was set to log at each axis continuously at 100 Hz with a sensitivity of ±8 g. The collar with the sensors attached was worn in addition to the dogs normal collar and tightened such that two fingers could be placed in-between the material and the neck (as recommended in [21]). Prior to fitting to collars the accelerometer was clapped against a hand in view of the video camera to place a distinctive signal in the data that would enable subsequent synchronisation between the accelerometer signal and the video. For the duration of each walk, all dogs were kept on lead and walked by the same handler to ensure route adherence and appropriate position for filming. Walking speed of the experimenter was approximately constant (~ 6.4kph) and continuous. As dogs were of different sizes the step frequency did vary between dogs. No attempt was made to control the gait of the dog, the side of the handler it walked on, or position relative to the handler (in front or behind). At the end of the walk, the instrumented collar was removed and the data was extracted for processing.

### Algorithm- step counting

**Fig. 3:**
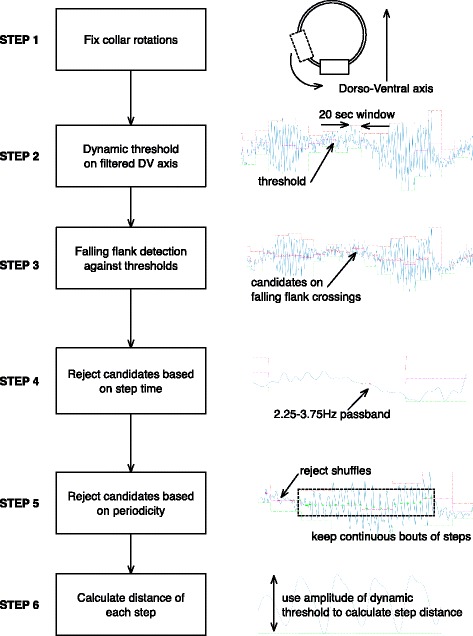
Flow diagram highlighting individual process stages of the algorithm for step counting

The algorithm begins by re-orientating the tri-axial accelerometer data to remove any effects of collar rotations (Step 1 in Fig. 3). This step is achieved through a method derived from mathematical principles laid out in Whaba’s problem. First, rotation matrix is derived such that when applied to the source signal, approximates the gravitational vector optimally in the Dorso-Ventral axis; the target sensor position. The approach starts by filtering any low frequency movements via a 4th order, zero-phase, low-pass Butterworth filter with **ω**_**c**_ at 0.1 Hz. The resulting signal is then projected out of Cartesian space onto unit (1 g) sphere in polar coordinates. Signal components falling within a threshold of 0.2 g of the sphere surface are segmented out and labelled as candidates for rotation correction. To preserve signals that might arise from natural movements such as posture transitions, a sliding window of length 10 s is passed over the data. Any orientation transitions occurring within the window are excluded from the list of candidate collar shifts. The remaining elements in the signal are subjected to a cost function (eq. (1)) that aims to place gravity maximally in the Dorso-Ventral axis; orthonormal to gravity.

1 $$ J(R)=\frac{1}{2}\sum \limits_{k=1}^N\parallel {w}_k-{Rv}_k{\parallel}^2 $$

Where **ω**_k_ is the k^th^ 3-axis sample in the rotated reference frame, **ν**_k_ is the k^th^ sample 3-axis sample in the target reference frame and ***R*** is the rotation matrix between the present and target reference frames. When evaluating eq. (1), only rotations about the sagittal plane are considered; we do not consider the case the collar twists on its self. In this way, the resulting rotation matrix can be input to transform matrix (eq. (2)) to find the new corrected collar position.

2 $$ T=\left[\begin{array}{ccc}\cos \kern0.5em \theta & 0& \sin \kern0.5em \theta \\ {}0& 1& 0\\ {}-\sin \kern0.5em \theta & 0& \cos \kern0.5em \theta \end{array}\right] $$

The next part of the algorithm seeks potential step candidates. In this step the principles laid out in [27] are followed. Firstly, the Dorso-Ventral axis is filtered with a 4th order Butterworth low-pass filter of **ω**_**c**_ at 0.2 Hz. With the resulting signal, a window of 20 s (inspired from [31]) is used to derive a zero-threshold (Step 2 Fig. 3); with zero-line half way between maximal-peak and minimal-nadir. Each time the zero-line is crossed by the falling-flank, the crossing times are stored (Step 3 Fig. 3) as step candidates for further comparison against rejection criteria.

**Fig. 4:**
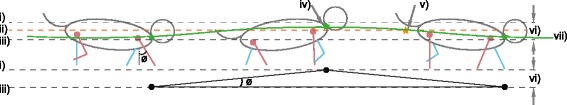
Illustration of approach used to find falling flank zero crossings and estimate step distance. Points marked are i) A_max,_ the peak acceleration observed in the dorsal-ventral axis; ii) (A_max_ - A_min_) /2, the mean acceleration observed in the dorsal-ventral axis; iii) A_min,_ the minimum acceleration observed in the dorsal-ventral axis; iv) collar mounted accelerometer; v) falling flank zero-crossing; vi) “bounce” calculated as A_max_-A_min_; vii) accelerometer signal aligned with ventral axis of dog (after re-orientation, filtering and smoothing). The figure shows the half angle of the step (*ϕ*) can be estimated from the trigonometry based around the bounce between steps. A_max_ and A_min_ can be used in eq. (4) for calculating step distance

The algorithm next establishes the time between each step-candidate is in a range prototypical of a head movement (Step 4 Fig. 3). As [31] describes, in quadruped locomotion, each stride (initial-contact to initial-contact on the same leg) will result in a dual-peak movement (Fig. 4). This can be modelled as simple harmonic motion around the scapulohumeral joint [32]. For the algorithm, this translates that each falling flank can be associated to a footfall (there is not necessarily alignment between the zero-crossing and initial-contact of the foot; see Fig. 4). The valid step frequency range was derived using eq. (3) as described in [33]. The heaviest and lightest animals in our cohort (64Kg Irish Wolf hound and 11.3Kg mixed breed), led to a step frequency range of 2.50–3.19 Hz. As a generalising step to accommodate dogs outside our cohort weight band, as well as ones that walk outside the ideal gait pattern, a margin was added and the valid step frequency range used for experiments was 2.25–3.75 Hz.

3 $$ stridefreq\left({\mathit{\min}}^{-1}\right)=269{W}^{-0.14} $$

The next part of the algorithm (Step 5 Fig. 3) is a sliding window containing 10 steps-candidates was used to filter out shuffling or non-periodic movements. Candidates that did not fall within *t*_*step*_ within each other were marked. Any window containing more than 3 marked candidates (< 70% of the window total) were rejected and did not contribute towards the final step count.

### Analysis- step counting

Videos were annotated by two observers using ELAN software (Max Planck Institute, Netherlands). The definition of one step was taken to be final-contact to initial contact for each thoracic limb. If the limb was not in full view of the camera, the step was not annotated. Steps were annotated wherever possible and no exclusions were made for cornering or for dog head posture. The claps inserted into the accelerometer and video were identified using manual inspection and time-offsets that allowed for synchronisation were calculated. Inter-observer reliability on manual step counts was tested with Cohen’s Kappa. The accuracy of the step counter was estimated as the percentage of steps correctly identified against annotations and the positive predictive value was calculated. True positive (TP) predictions were regarded as when the predicted step lay between the final and initial contact of an annotated step (swing phase). False positives (FP) were counted when a prediction was made but no aligning annotation was present. From TP and FP, Positive Predictive Value (PPV) was estimated. Pearson’s correlations and Bland Altman statistics were calculated for comparison of predicted steps against video annotations using MatLab R2016b.

### Subjects - distance travelled

For the distance experiments, data was gathered from 10 dogs diagnosed with osteoarthritis in at least one limb by their general veterinary practitioner. These dogs were a convenience sample from a larger group of dogs with osteoarthritis involved in another study. Breeds included: Mixed-breed, Collie, Labrador, Rottweiler, Springer Spaniel and Toy Poodle, aged 8–13 years (see Table 2).

**Table 2:** Details of subjects, Quantity (Qty) of usable walks, distance estimated using GPS and Distance estimated using an algorithm applied to an accelerometer signal. Each walk length is listed separated by a semicolon

ID	qty Walks	Weight (kg)	Breed	Sex^a^	dist GPS (m)	dist Algorithm (m)
D1.1	2	NA	Mixed	MN	2590; 2430	3307;3355
D1.2	3	NA	Mixed	MN	732; 878; 699	953;957;974
D1.3	1	NA	Mixed	FN	577	712
D1.4	2	NA	Mixed	MN	815;568	847;579
D1.5	2	20	Labrador	FN	852;1310	609;812
D1.6	1	13	Toy Poodle	MN	921	1446
D1.7	1	17	Springer Spaniel	FN	1830	2853
D1.8	1	34	Rottweiler	FN	776	851
D1.9	1	15	Collie	MN	1330	1240
D1.10	1	17	Collie Cross	MN	650	688

^a^For sex *F* Female, *M* Male, *N* neutered

### Experiment- distance travelled

Owners of these dogs were asked to keep to their normal routine for one week (which could commence on any day of the week or weekend and could include exercise of any type). Each dog was fitted with a soft weave nylon flat collar instrumented with a G-Paws GPS (G-Paws Limited, UK) and accelerometer (VetSens UK). This instrumented collar was worn in addition to the dog’s normal collar and tightened such that two fingers could be placed in between the collar and the neck. The accelerometer was attached to the outside of the collar using a single layer of Gorilla Tape and the G-Paws was attached using the provided housing. The accelerometer was set to record continuously for a period of seven days with a sampling frequency of 100 Hz at ±8 g sensitivity. Owners were requested to use the G-Paws only during walks and to keep the devices plugged in to be charged when not in use. Owners were also asked to keep a diary of walk times and locations.

### Algorithm- distance travelled

Estimating distance from step counts can be done in the most basic way by first estimating stride length (directly from weight as suggested in [33]) and then multiplying by the number of strides. This simplistic approach has been shown to falsely estimate distance in field-based experiments [34], as stride length changes with gait, terrain and incline. A more sophisticated approach is to estimate stride length dynamically by means of a gait model. For this, the dog’s walking gait (which makes up the majority or transportive-locomotion according to [1]) was split as two independent sets of legs; pelvic and thoracic [32]. Each pair is then modelled as an inverted-pendulum with the thoracic centred on the scapulohumeral joint and pelvic centred on the coxofemoral joint. Over the course of a stride, each set works by transferring the centre of mass (COM) on to the other pair. The accelerometer mounted ventrally on the neck is sensitive to the undulating movement of the COM through the stride and schematically depicted in Fig. 4.

Using trigonometry, *h is can be derived as* eq. (4)

4 $$ h=\sqrt[4]{{\mathrm{A}}_{max}-{\mathrm{A}}_{min}} $$

where *A*_*max*_ and *A*_*min*_ are the maximum and minimum values of ventral acceleration (in *m/s*^*2*^) observed around the zero-crossing). For distance estimation, each step is considered independently and stride distance is approximated as the sum of two consecutive steps.

### Analysis- distance travelled

The raw files recorded on the G-Paws sensors were manipulated using Google Earth software v7.1. This software facilitates walks to be manually segmented according to a rule-base described in [35] which suggests maximal and minimal traveling velocities. For each walk, the total distance travelled was calculated and walks of less than 100 m were rejected. Owner diaries were used to confirm the segmented walks occurred on the correct day and time, had a legitimate duration, and walk location corroborated the GPS data. Using the start and end times directly from the GPS data segments, time-aligned accelerometer data was extracted using a custom Matlab script. The distance estimation mean similarity was estimated by calculating the mean of 1- the percentage difference between GPS and algorithm estimated distance for each walk. Pearson’s correlations and Bland Altman statistics were calculated for comparison against the GPS distance estimate using Matlab R2016b.

## Results

### Step counting

**Fig. 5:**
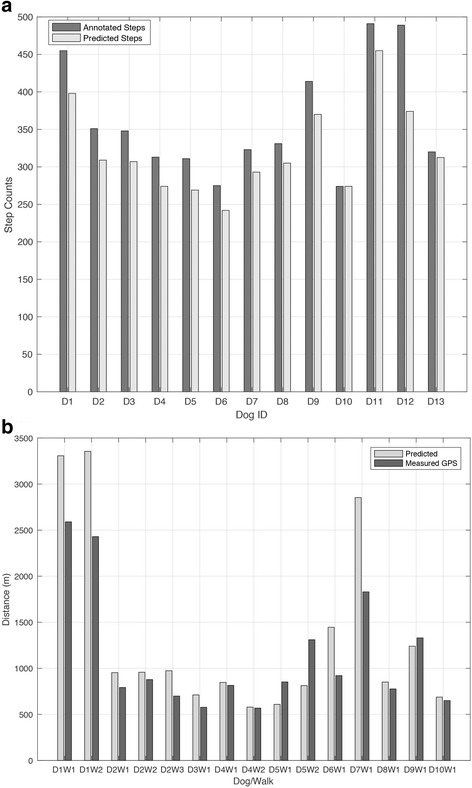
**a** Predicted steps from algorithm to counted steps from annotated video footage for each dog; **b** Estimated distance from algorithm to distance measured via GPS for each walk

All the dogs recruited for this part of the experiment (*n* = 13) were healthy adult dogs. The cohort had a mean age of 5.7 years and were made up of 9♀ and 4♂. Details for each dog are included in Table 1. Each dog tolerated the accelerometers well and completed the walk successfully and in total 4695 steps were annotated from the video (mean of 361 per dog with stdev of 75.8). The pedometer algorithm was able to detect 91.0% of these with a PPV of 0.98. The predicted and annotated step counts were highly correlated (*r* = 0.99, *p* = 6.072e-10) and Bland Altman statistics revealed a mean difference of 3.96 steps and a critical difference of 25 steps, with all but one dog within the critical difference. Distributions of steps for each dog were relatively equal (Table 1, Fig. 5a).

### Distance travelled

All dogs for this part of the study (*n* = 10) had been previously diagnosed with osteoarthritis (see Table 2 for subject details). The cohort was made of 4♀ and 6♂. Over the course of the week, all dogs recorded GPS data from at least 1 walk, with 3 dogs completing 2 walks and 1 dog completing 3. The GPS based walk distance ranged from 0.57–2.59 km (mean 1.1 km). All dogs tolerated the equipment well and in total, the GPS equipped collars logged 20,184 m meters over 15 individual walks. This distance was made up of a roughly equal distribution of on and off-lead time (based on owner self-reports). The mean similarity between GPS and algorithm estimated distances was 79% with stdev of 16 (see Table 2, Fig. 5b). The predicted and GPS measured distances were highly correlated (*r* = 0.98, *p* < 0.001) and Bland Altman statistics revealed a mean difference of -211 m and a critical difference of 699 m, with all but one walk within the critical difference.

## Discussion

Here we present two algorithms which show good promise in being able to detect steps and estimate distance travelled by a dog, based on data extracted from a collar-mounted accelerometer. Due to the distance estimates being derived from accelerometer signals, rather than GPS, they are supportive of indoor based measurements (where GPS coverage is unfeasible), as well as long-term, continuous operation (not possible with GPS technologies due to high power consumption). The algorithm described is a first step in an accurate tailored system for measuring dog walking out of laboratory conditions. It not specific to any hardware design and with minimal modification (perhaps just axis rotation), could be used on data arising from either existing research grade hardware or raw tri-axial data available from consumer grade devices. Furthermore, unlike [28, 36], we did not find any observable differences between accuracy of distance estimates and dog size, although the sample in the current study was relatively small. In [28, 36] pedometers were used which were designed for human gait and unlike the approach used here were not tailored for the canine gait. Further research is needed to establish whether accuracy of pedometer devices is influenced by weight and not just size as has been found in humans [37]. This is important because it could affect research findings. For example, the association observed between body condition score and steps walked in [28, 36] may have been influenced by a relationship between pedometer accuracy and body weight. The ability to calculate the number of steps taken in time and the distance travelled for steps have two clinically relevant potential uses. Firstly, temporal and spatial measurements of ambulatory behaviour, which can be measured with accelerometers and the algorithms outlined in this paper, alter with movement disorders or other diseases which effect the motor system. The methods presented here therefore offer the first step towards automatic diagnostics and monitoring for veterinarians, owners or researchers interested in disease prediction. Secondly, the number of steps taken per day or distance travelled are simple metrics which are familiar to the general public (e.g. public health schemes promoting 10,000 steps a day). Thus, measuring number of steps or distance travelled could be used to support promotions of healthy lifestyles for dogs, or for weight loss interventions.

The number of steps estimated was highly accurate, but there were a small number of occasions where it was less accurate. By manually reviewing video footage of when accuracy was lower, we found these mostly occurred when a dog was excessively pulling on the leash (such that the leash was taking the majority of the dog’s weight). The other occurrence observed when the algorithm failed to perform well, was when dogs walked in tight circles, such to manoeuvre onto a preferred side of the dog walker. If such a movement was classified as a non-step it was rejected. As the algorithm uses a windowing approach, there is the possibility a complete window of steps is rejected due to it containing a single poor candidate step. This could be one reason for the approach consistently underestimating step counts. Despite the algorithm not being able to detect left from right steps, it performed without bias in their delineation. It is anticipated that addition of gyroscope data to the accelerometer data could yield left-right detection; as it has done in human studies [38].

In this study accelerometers and GPS collars were fitted to a second collar, if the dog already wore one as standard. This was to prevent changes in rotation of the collar or other forces caused directly by an attached leash. No owners reported any concerns with their dog wearing two collars, even when asked directly and dogs showed no visible signs of discomfort. All dogs in our sample were walked on a leash rather than a harness, however, we foresee no reason why the combination of an instrumented collar and harness would influence the algorithms proposed.

The subjects of this study were biased towards the medium to large breed range. Despite this, it did contain a mixture of healthy and arthritic dogs as well as one chondrodysplastic breed (Welsh Corgi). This dog did not appear as an outlier in the dataset. However, to thoroughly characterise the role of morphology, body condition score (obesity) and size, a subsequent study is required. Furthermore, theoretically the inverted pendulum model used to estimate step distance should not be affected by disease such as osteoarthritis, a further study of distance measurements on healthy dogs would confirm this.

During both step count and distance experiments, no effort was made to standardise the gait of the dog. The study design used here meant it was not possible to precisely determine the effect of gait on detection performance or mitigate it through design. Conversely, the data set could be considered naturalistic and thus the results presented are representative of performance in a real deployment. Furthermore, in the distance experiment, the owner determined the dog pace, duration and distance of the walk and put on the collar. In the GPS based data there was an obvious outlier in one walk. While it is not possible to precisely pinpoint the cause of the outlier, the authors suspicion is that this related to collar attachment. This highlights the need for clear instructions for collar placement or that instrumentation is carried out by trained clinicians to ensure proper attachment; highlight by [21] as important.

## Conclusions

The experimental results show promise that the approach is suitable for accurately discerning steps and associated distance from a collar mounted accelerometer. The use of accelerometers and these algorithms may be preferable to alternative devices such as GPS, due to their suitability for extended durations and in both indoor and outdoor environments. The approaches presented are also preferable to those designed for humans or from dog specific commercially available devices. The reason for this is because humans have a lower cadence than dogs and are obviously bipedal as opposed to quadrupedal; devices designed for humans are not ideally suitable for dogs. Furthermore, commercial devices for pets which may be specialised for dogs have used closed source approaches. Publishing these approaches allows for comparison between devices, without continuous revalidation for each new model of a device. Knowing the rate and number of steps or the distance travelled of a dog could be useful clinically for monitoring disease, or for weight loss monitoring programmes.

## Abbreviations

COM: Centre of mass
FP: False positive
GPS: Global positioning system
PPV: Positive predictive value
TP: True positive

## References

[1] Johnston SA (1997). Osteoarthritis: joint anatomy, physiology, and pathobiology. Vet Clin North Am Small Anim Pract Elsevier.

[2] Belshaw Z (2016). Decision making and welfare assessment in canine osteoarthritis.

[3] Belshaw Z, Asher L, Dean RS (2016). Systematic review of outcome measures reported in clinical canine osteoarthritis research. Vet Surg Wiley Online Library.

[4] Belshaw Z, Asher L, Harvey ND, Dean RS (2015). Quality of life assessment in domestic dogs: an evidence-based rapid review. Vet J.

[5] Robertson ID (2003). The association of exercise, diet and other factors with owner-perceived obesity in privately owned dogs from metropolitan Perth, WA. Prev Vet Med.

[6] Cheung KW, Starling MJ, McGreevy PD (2014). A comparison of uniaxial and triaxial accelerometers for the assessment of physical activity in dogs. J Vet Behav Clin Appl Res.

[7] Clarke N, Fraser D (2016). Automated monitoring of resting in dogs. Appl Anim Behav Sci.

[8] Clark K, Caraguel C, Leahey L, Béraud R (2014). Evaluation of a novel accelerometer for kinetic gait analysis in dogs. Can J Vet Res.

[9] Michel KEK, DCD B (2011). Determination and application of cut points for accelerometer-based activity counts of activities with differing intensity in pet dogs. Am J Vet Res.

[10] Gerencsér L, Vásárhelyi G, Nagy M, Vicsek T, Miklósi A (2013). Identification of behaviour in freely moving dogs ( Canis familiaris ) using inertial sensors. de Polavieja GG, editor. PLoS one. The Japanese Society of Veterinary. Science.

[11] Michel KE, Brown DC (2014). Association of signalment parameters with activity of pet dogs. J Nutr Sci.

[12] Morrison R, Reilly JJ, Penpraze V, Pendlebury E, Yam PS (2014). A 6-month observational study of changes in objectively measured physical activity during weight loss in dogs. J Small Anim Pract.

[13] Whistle GPS Pet Tracker for dogs and cats. Available from: https://www.whistle.com/. [cited 8 Aug 2017].

[14] Heyrex Pet Tracker. Available from: http://www.heyrex.com/en/?. [cited 8 Aug 2017].

[15] PetPace Pet Monitor. Available from: http://petpace.com/. [cited 8 Aug 2017].

[16] Pod GPS Tracker. Available from: https://www.podtrackers.com/. [cited 8 Aug 2017].

[17] Iota Tracking device. Aveailable from: http://www.iotatracker.com/. [cited 8 Aug 2017].

[18] Tractive® GPS device for dogs, cats and other pets. Available from: https://tractive.com/en/. [cited 8 Aug 2017].

[19] FitBark Dog Activity Monitor. Available from: https://www.fitbark.com/. [cited 8 Aug 2017].

[20] Yashari JM, Duncan CG, Duerr FM (2015). Evaluation of a novel canine activity monitor for at-home physical activity analysis. BMC Vet Res.

[21] Martin KW, Olsen AM, Duncan CG, Duerr FM (2016). The method of attachment influences accelerometer-based activity data in dogs. BMC Vet Res.

[22] Olsen A, Evans R, Duerr F (2016). Evaluation of accelerometer inter-device variability and collar placement in dogs. Vet Evid.

[23] Preston T, Baltzer W, Trost S (2012). Accelerometer validity and placement for detection of changes in physical activity in dogs under controlled conditions on a treadmill. Res Vet Sci.

[24] Ladha C, Jackson D, Ladha K, Olivier P (2013). Open movement: an open source sensor platform. Github repos.

[25] Bravata DM, Smith-Spangler C, Sundaram V, Gienger AL, Lin N, Lewis R (2007). Using pedometers to increase physical activity and improve health. JAMA. J Am Med Assoc.

[26] Levine JA, Mecrady SK, Lanningham-Foster LM, Kane PH, Foster RC, Manohar CU (2008). The role of free-living daily walking in human weight gain and obesity. Diabetes.

[27] Weinberg H. Using the ADXL202 in pedometer and personal navigation applications. Analog Devices AN-602 Appl note. 2002:1–8.

[28] Warren BS, Wakshlag JJ, Maley M, Farrell TJ, Struble AM, Panasevich MR (2011). Use of pedometers to measure the relationship of dog walking to body condition score in obese and non-obese dogs. Br J Nutr.

[29] Ladha C, O’Sullivan J, Belshaw Z, Asher L (2017). GaitKeeper: a system for measuring canine gait. Sensors. Multidisciplinary Digital Publishing Institute.

[30] Kilkenny C, Browne WJ, Cuthill IC, Emerson M, Altman DG (2012). Improving bioscience research reporting: the ARRIVE guidelines for reporting animal research. Osteoarthr Cartil.

[31] Ying H, Silex C, Schnitzer A (2007). Automatic step detection in the accelerometer signal. 4th Int. work. Wearable implant. Body Sens Networks.

[32] Lee D, Bertram J, Todhunter R (1999). Acceleration and balance in trotting dogs. J Exp Biol.

[33] Heglund NC, Taylor CR, McMahon TA (1974). Scaling stride frequency and gait to animal size: mice to horses. Science.

[34] Buckley CMF, Colyer A, Skrzywanek M, Jodkowska K, Kurski G, Gawor J (2011). Use of pedometers to measure the relationship of dog walking to body condition score in obese and non-obese dogs. Br J Nutr.

[35] Carlson JA, Jankowska MM, Meseck K, Godbole S, Natarajan L, Raab F (2014). Validity of PALMS GPS scoring of active and passive travel compared with SenseCam. Med Sci Sports Exerc.

[36] Chan CB, Spierenburg M, Ihle SL, Tudor-Locke C (2005). Use of pedometers to measure physical activity in dogs. J Am Vet Med Assoc.

[37] Crouter SE, Schneider PL, Bassett DR (2005). Spring-levered versus piezo-electric pedometer accuracy in overweight and obese adults. Med Sci Sports Exerc.

[38] McCamley J, Donati M, Grimpampi E, Mazzà C (2012). An enhanced estimate of initial contact and final contact instants of time using lower trunk inertial sensor data. Gait Posture.

